# Chemical Investigations on the Blood in the Neuroses

**Published:** 1849-01-01

**Authors:** 


### CHEMICAL INVESTIGATIONS ON THE BLOOD IN THE NEUROSES.

*A Memoir presented to the Academy of Sciences at Paris, on the 29th of November,* 1847, *by* DR. MICHÉA.

(*Continued from* No. III.)

### THIRD PERIOD.

*Insanity—Absence of maniacal agitation, and ambitious monomania—Imperfect general paralysis—Involuntary excretions—Augmentation of the globules—Diminution of the albumen.*

CASE 1.—M——, a hatter, aged 38, of a robust constitution, and a sanguineous temperament; has been at Bicêtre from the month of December, 1846.

On the 25th of June, 1847, he presented the following condition. He answered correctly all questions addressed to him on his age, his profession, and family, but was unable to indicate the day of the week, the present month, or the year. Easily excited to tears on any mention of his wife and children; absence of agitation, and of all delirious ideas regarding fortune or grandeur.

Marked embarrassment of pronunciation, trembling of the lips and hands; tottering gait; good appetite; involuntary excretion of urine and fæcal matter. The urine not rendered turbid by heat or on the addition of some drops of nitric acid.

26th.—Standing was almost impossible. Four hundred grammes of blood ordered to be drawn.

27th.—The same state as the evening before; the pulse strong and full, seventy-six beats in a minute. Three hundred grammes of blood drawn.

The blood taken on this last occasion has alone been examined.

*Analysis of* 1000 *parts of blood.*

| | | |
|---|---|---|
| Water | | 794·142 |
| Globules | | 138·414 |
| Fibrin | | 3·658 |
| Solid matters of the serum | organic | 53·453 |
| | inorganic | 10·333 |

The number 138 which here represents the quantity of the globules, is below what it would have been if four hundred grammes of blood had been drawn twenty-four hours before that which was made the object of our analysis. Although a little too low from this same reason, the number representing the organic materials of the serum is nevertheless absolutely low, independently of the first venesection. It is further to be remarked that the urine does not contain any trace of albumen, and that the diminution of this principle of the blood was not accompanied by any form of dropsy.

*Feebleness of memory—Maniacal excitement—Incoherence of ideas, with predominance of ambitious monomania—General paralysis—Involuntary excretions—Slight diminution of albumen.*

Case 2.—S——, a clerk, aged 40, of a robust constitution, and sanguineous temperament. Has been at Bicêtre since the month of June, 1846.

At the present time (10th of June, 1847) he is in the following condition: very considerable loss of memory, being unable to remember his name or age; strongly marked loquacity; incoherence in his speech, with a predominance of ambitious ideas; he declares that *he has the little Buonaparte in his knee* (*dans son genou*); *that he had humbugged his father; is rich; has the monopoly of all the white bread and truffles and Thorins wine consumed in Paris.* He appears happy; says he has good health and a good digestion. Occasionally he sings.

Much embarrassment in his pronunciation, trembling of the upper and lower extremities; involuntary excretion of urine; voracious appetite, and an incessant wish to indulge it. Pulse of ordinary strength, 72 in the minute. Two hundred grammes of blood drawn.

*Analysis of* 1000 *parts of blood.*

| | | |
|---|---|---|
| Water | | 804·439 |
| Globules | | 125·612 |
| Fibrin | | 2·634 |
| Solid matters of the serum | organic | 58·212 |
| | inorganic | 9·103 |

*Insanity — Absence of ambitious monomania — Imperfect general paralysis — Involuntary excretions — Convulsions and epileptic attacks — Fever — Death — The meninges adhering to the cerebral convolutions — Injection of the vessels of the pia-mater — Abundant serosity at the base of the cranium, in the lateral ventricles, and in the cellular sub-arachnoid tissue, the gray matter abounding in red specks — Diminution of the water — Augmentation of the fibrin, and of the solid matters of the blood.*

CASE 3.—Lourmant, aged forty-two, entered Bicêtre in 1845. Is of a strong constitution, and sanguineous temperament; and has all the characteristics of *embonpoint.*

Embarrassment of speech; standing and walking accompanied by tottering; excessive trembling of the upper extremities. Calm, but manifesting feebleness of memory with respect to events of recent date; absence of delirious ideas concerning fortune and greatness; involuntary discharge of urine and fæces; voracious appetite.

On the 17th of May, 1847, sudden loss of consciousness; convulsive epileptic movements of the whole body, but principally of the muscles of the face. (Venesection prescribed.) The 18th, the patient makes no reply to the questions asked him; continued spasmodic convulsions, which recur every four or five seconds, and are always more marked in the face than in the other parts of the body; a supine posture is adhered to; there is no voluntary movement, and no sign of sensibility when the skin is pinched; hiccup; the face, neck, and limbs are purplish red; the pulse moderately full, a hundred in a minute. 150 grammes of blood were drawn during one of these epileptic attacks.

The 19th, he died.

*Appearances after Death.*—The brain appeared to escape on cutting through the cerebral membranes, which adhered to the convolutions at several points. The layer of arachnoid which covers the inner surface of the hemispheres and their convexity was raised up by serosity. Below this membrane the pia-mater was infiltrated with a great quantity of limpid serosity, which flowed forth at all parts. The encephalic vessels were injected. The lateral ventricles and the base of the brain were full of serosity. There was no ramollissement, but a decided excess of red specks of the gray matter.

The blood drawn at the second time of venesection was alone analyzed.

*Analysis of* 1000 *parts of blood.*

| | |
|---|---|
| Water | 755·58 |
| Globules | 119·98 |
| Fibrin | 10·75 |
| Solid matters of the serum | 113·69 |

*Insanity—Absence of ambitious monomania—Involuntary excretions —Slough on the sacrum—Voracity—Febrile movement—Cerebral congestion and convulsions—Augmentation of the globules and of the fibrin.*

Case 4.—Roubaud, a carpenter, aged 40, of a robust constitution, and a bilio-sanguineous temperament.

He has been at Bicêtre since July, 1846.

In the month of June of the year 1847, he presented the following condition :—has forgotten almost all the events of his life, recent events leave no impression on his mind. Absence of delirious ideas respecting fortune and greatness. Pronunciation much embarrassed ; walking and standing attended by difficulty ; calmness ; considerable appetite ; incipient slough on the sacrum ; involuntary excretions.

On the 18th, the patient suddenly lost all consciousness ; his face became red, and the eyes convulsed; perfect insensibility of the skin; contractions of the muscles; heat of the skin; the pulse 120 in a minute.

300 grammes of blood were drawn.

*Analysis of 1000 parts of blood.*

| | |
|---|---|
| Water | 743·071 |
| Globules | 179·728 |
| Fibrin | 4·890 |
| Solid matters of the serum | 72·311 |

The considerable augmentation of the globules corresponds also in this case with the incontestable existence of cerebral congestion, unaccompanied by any trace of ambitious monomania. The increase in the number of the fibrin is explained by the manifestation of an acute phlegmasia of the cerebral substance, which is shown by the spasmodic condition of the muscles of the eye, by the contraction of the tendons, &c.

Thus we find that, in 16 persons, more or less insane, and affected by general paralysis, without any distinction of degrees, the *globules* were in five cases above their physiological limits, the minimum being 148, and the maximum 179; in five cases below those limits, the minimum being 32, and the maximum 108. In six cases they oscillated between their normal limits from 138·8, 138·4, 125·02, 125, 120, 119. From these six cases we must, however, except two, in one of which the abstraction of 400 grammes of blood, drawn in the first venesection on the preceding evening, had lowered the number of the globules to 138·4; whilst in the other, the abstraction of 700 grammes of blood, likewise on the preceding evening, had lowered the number of this principle of the blood to 138·8; so that in reality these two last-named cases come within the category of those in which the globules experienced an augmentation: thus making the number of such cases seven instead of five.

The *fibrin* was below its physiological limits in four cases; the highest number was 1·92, and the lowest 1·44. It rose in two cases above those limits (4·89, 10·7). It oscillated in ten cases between these physiological limits (2·476, 2·56, 2·62, 2·63, 2·07, 2·99, 3·04, 3·25, 3·653, 3·658). The absolute decrease corresponded in two cases, with a diminution of the globules, and in two cases with their augmentation. Finally, in four cases the diminution was relative—that is to say, the number of this principle of the blood remained normal, whilst the number of the globules rose above its physiological limits.

The *albumen* was found in five cases much below its mean proportion; the maximum was 58, and the minimum 40. The *solid matters of the serum* exceeded in three cases their mean quantity; the minimum was 105, and the maximum 113. In eight cases these matters did not vary notably from their mean (89, 88, 85, 82, 79, 71, 71, 64).

The *water* exceeded its mean proportion in nine cases; the minimum was 794, and the maximum 879. It fell seven times below these limits, the maximum being 778, and the minimum 743.

Before we attempt to draw from these chemical facts any inductions capable of throwing light on the pathological physiology and the treatment of general paralysis in the insane, it will be necessary briefly to draw attention to certain propositions recently enounced in hematology as applied to pathology, and which form a part of the laws of humorism as at present established.

According to MM. Andral and Gavarret, augmentation of the globules and absolute or relative diminution of the fibrin (whether one alone of these principles of the blood vary, or whether both simultaneously change their proportion,) characterize *cerebral congestion* and *hæmorrhages* in a great number of cases. On the other hand, diminution of the globules is the cause of anæmia, and an excess of fibrin precedes invariably the development of acute phlegmasia. Finally, the diminution of the albumen would appear to correspond with the existence of a certain number of dropsical affections.

The data yielded by nine cases of general paralysis of the insane exhibit determined cerebral congestion in four cases, with one case of simple tendency towards that affection; all these being cases in which I discovered in the blood, either simultaneously or independently of one another, an augmentation of the globules, and an absolute or relative diminution of the fibrin, and where the congestion was characterized by the following symptoms: injection and turgescence of the face, sudden inability to walk or stand upright, and hemiplegia disappearing under the influence of blood-letting at the end of twenty-four or forty-eight hours. Congestion here corresponded in one case to an isolated augmentation of the globules, and in three cases with the latter combined with relative diminution of the fibrin; finally, an absolute diminution of the latter principle of the blood was only manifested, accompanied by the simple tendency to cerebral congestion.

These facts confirm, as we see, the laws of pathological hematology laid down by MM. Andral and Gavarret; and prove, that in the majority of cases, the excess of the globules, and the absolute or relative diminution of the fibrin, are either independently of one another, or simultaneously, the immediate cause of cerebral congestion. It must, however, be observed, that in accordance with our analyses, the modification of the proportion of the globules appears to have more influence than the modification of the fibrin on the development of this disease; since, in the four cases of well-determined cerebral congestion, the former of these two elements of the blood was constantly found to have experienced an absolute augmentation, contrary to the second, whose diminution was merely relative, the number never falling below the physiological limits.

These results are, on this very account, different from those obtained by M. Erlenmeyer, who is of opinion that cerebral plethora in the insane is always accompanied by a diminution of the globules, although a less considerable one than that existing in chlorosis; and hence he concludes that blood-letting should be discarded as a therapeutic agent in this malady.

Symptoms of convulsions, alternating with cataleptic attacks, were connected in one instance with a diminution of the globules.

Phenomena characteristic of acute cerebral phlegmasia coincides in two cases with an augmentation of the numbers of the fibrin.

Finally, in five cases in which the albumen fell considerably below its mean proportion, there was no trace of dropsical effusion in the cellular tissue, the peritoneum, the pleuræ, pericardium, or tunica vaginalis. How are we to explain such a diminution of the albumen of the blood in these five cases? There was no loss of albumen by the urine from any inflammatory affections of the kidneys, for, on being analyzed, it did not become turbid under the action of heat, alcohol, or nitric acid. May not some influence have been exercised by the effusions of serosity into the ventricles of the brain, and into the sub-arachnoid cellular tissue, since such effusions are alike copious and frequent, especially when general paralysis has progressed to the latter periods of its course? Might not a quantity of albumen have been found in the cerebral serosity—I will not say sufficient to balance the diminution of this principle in the blood, since this cannot take place in any instance, as has been proved by M. Andral—at any rate equivalent to a considerable portion of this diminution? This theory might present some probability, judging by analogy, since, in twenty-two analyses of serosity taken from the peritoneum, pleuræ, pericardium, cellular tissue, and the tunica vaginalis, M. Andral found, for 1000 parts of fluid, that the albumen was represented by the numbers 59, 55, twice by 51, 49, 48, 47, 41, 40, 35, 30, 28, 19, 15, 14, twice by 12, 11, 10, and three times only by a number less than the last named, but never below 4. It would appear, from less recent analyses than our own, that the arachnoid serosity was much less rich in albumen, and sometimes, indeed, contained no more than scarcely appreciable traces of this principle. The serous fluid contained in this membrane does not coagulate under the action of alcohol, heat, or acids, according to the statement of M. Haldat.* Its specific gravity differs but little from that of ordinary water, it is not viscid, and froths but little on being agitated in the air. 100 parts of this fluid yielded—

| | |
|---|---|
| Water | 96·5 |
| Muriate of soda | 1·5 |
| Albumen | 0·6 |
| Mucus | 0·3 |
| Gelatine | 0·9 |
| Phosphate of soda (quantity undetermined) | |
| Phosphate of lime (conjectural) | |

Although the number of albumen is here so low (six parts to 1000 of serosity), it is still lower in another analysis made by Dr. Marcet. In 1000 parts of arachnoid serosity, this chemist found the following proportions :—

| | |
|---|---|
| Water | 990·8 |
| Muco-extractive matter with albumen | 1·12 |
| Muriate of soda | 6·64 |
| Sub-carbonate of soda, with a small portion of some other alkaline sulphate | 1·24 |
| Phosphates of lime, magnesia, and iron | 0·2 |

A fact worthy of notice, and one which proves that, in the study of vital phenomena, conclusions drawn from observations made on animals ought not always to be applied to man, is afforded by Professor J. F. John, whose analysis of the arachnoid serosity, in a child who died of hydrocephalus, confirms the assertions of Marcet and M. de Haldat, for this writer affirms that he has met with a large quantity of albumen in the serosity emanating from the cerebral ventricles of calves killed in slaughter-houses. The author of the article "*Sérosité*," of the *Diction-*

* See a report of Professor Deyeux, in the "Bulletin de la Faculté de Médecine de Paris, et de la Société de Médecine," année 1814, No. VI., p. 125.

*naire des Sciences Medicales*, asserts, moreover, that he is in the possession of observations corroborative of this fact in comparative chemistry.

Results which tended to establish such an exception, in the human subject, to the law which governs the composition of the fluid emanating from all the other serous membranes, were well deserving of a repetition. The motives that urged me to attempt to verify these results arose from the fact, that the inquiries of MM. Marcet, Haldat, &c., were exclusively directed to the serosity taken from the arachnoid of children who had died of hydrocephalus. Now, it appears to me to be absolutely necessary to determine whether the composition of the arachnoidal fluid is the same in adults as in children, before we can venture to generalize and conclude, as M. Haldat has done, that the serous fluid contained in the peritoneum, pleuræ, pericardium, tunica vaginalis, and the meshes of the cellular tissue, differs almost entirely from that which moistens the membranous envelopes of the brain and of the spinal cord, and that albumen and soda predominate in the former, and muriate of soda and gelatine in the latter.

In a man aged 28, who died of meningeal encephalitis, I removed about three teaspoonfuls of slightly rose-coloured serosity from the lateral ventricles. This fluid, on being carefully separated from the blood with which it was mixed, was rendered sensibly turbid by the action of alcohol, heat, and nitric acid. In a woman aged 60, who had died of pulmonary tuberculosis, I obtained about 16 grains of purer cerebral serosity. This fluid likewise coagulated when treated with the re-agents already named. The following were the results yielded by an analysis of 1000 parts of the fluid :—

| | |
|---|---|
| Water . . . . . . . . . . . . . . . | 991 |
| Salts . . . . . . . . . . . . . . | 2 |
| Albumen and extracto-mucous matters . . | 7 |

It follows, as we see, from these analyses, that the albumen contained in the cerebral serosity is appreciable by ordinary re-agents, in opposition to that which M. Haldat asserts to be the case in hydrocephalic children, but that this organic principle is found in much less considerable quantities than in the serosity of the peritoneum, pleuræ, pericardium, and tunica vaginalis.

Hematology proves, on the other hand, that there may be a spontaneous diminution, or rather an insufficient formation, of the albumen of the blood in nearly one-third of all the cases of general paralysis. I say a spontaneous diminution and an insufficient formation, for if the albumen had at first occurred in its normal quantity, and afterwards escaped from the blood, it must have been found in the urine, and to a certain degree in the serosity of the cellular tissue of the peritoneum, &c., as we find to be the case in Bright's disease, and in dropsical affections consequent on organic lesions of the heart. The urine was not rendered turbid by heat, alcohol, or nitric acid, nor were there in any case traces of œdema, anasarca, ascites, &c. On the other hand, the albumen contained in the encephalic serosity, which, according to M. Bayle, fills more or less entirely the lateral ventricles in two-thirds of the patients in the last stage of general paralysis, and in the cases of one-third of the whole number distends these ventricles beyond measure, and dilates them to so great a degree as to constitute chronic hydrocephalus, is still less able to represent the quantity of albumen removed from the blood; for we find from analysis that the arachnoid serosity contains infinitely less albumen than the serosity occurring in other serous cavities.

We will now proceed to compare the results yielded by chemistry with those presented by pathological anatomy, and consider the differences and resemblances existing between them.

According to M. Bayle—1. Cerebral congestion constantly precedes, with a more or less rapid course, general paralysis, and is therefore its proximate or direct cause.

2. Paralysis is owing to compression of the brain, induced by sanguineous congestion, and, in one eighth of the cases, is accompanied by a sanguineous effusion between the meninges.

3. The excessively violent and continual agitation is often induced by an intense inflammatory action, which gives rise to an albuminous exudation on the surface of the arachnoid, consisting occasionally of small masses of a yellowish, greyish, or whitish exudation, but which is generally more abundant, becoming transformed into false membranes, analogous to those frequently met with on the pleura, pericardium, peritoneum, &c.

4. The epileptic attacks, partial or general trembling, *subsultus tendinum*, convulsions, grinding of the teeth, rigidity, tetanic contractions, and tremblings accompanied by contractions, depend on the inflammation of the grey matter, subsequent on chronic inflammation of the meninges.

5. The apoplectic attacks so frequent in the third period are almost always induced by a sudden congestion of the vessels of the pia-mater and of the brain, very rarely by an afflux of serous fluid, and in no case by cerebral hæmorrhage.

6. In the third period, the cessation of the agitation, the augmentation of the paralysis, and the insanity, are the signs of a compression of the brain, which depends on an exudation of serosity in the cavity of the arachnoid, a serous infiltration of the pia-mater, and of an effusion of a similar nature into the lateral ventricles.

7. The state of stupidity, with an obliteration of the faculties and ideas, and general paralysis of almost the whole body, are results of compression of the brain, and consequently of the highest degree of serous effusion. Chemistry, as well as pathological anatomy, assigns a considerable influence to cerebral congestion in madness and paralytic insanity, since, in the majority of cases, (in nine cases out of sixteen,) the blood of patients affected by the species of madness now under consideration presents those alterations in the proportion of its principles which Messrs. Andral, Gavarret, and other hematologists, have given as the characteristics of the first of these affections—viz., an augmentation of the globules, and a diminution of the fibrin, whether the modification affect only one or both of these principles simultaneously. We do not, however, agree with M. Bayle in believing that the tendency to the flow of blood in the encephalic vessels exists in all persons affected by general paralysis, and that it constantly precedes its occurrence; in other words, that it is the immediate or direct cause of the affection; for, according to this hypothesis, every cerebral congestion would sooner or later, necessarily and infallibly, produce general paralysis, which is contrary to the results of daily observation. It is a main condition, but not a sufficient cause. This admits of ready explanation. General paralysis especially supervenes in the prime of life, from the age of thirty to fifty. The male sex, and persons having a sanguineous temperament, and a robust and athletic constitution, are most liable to this affection. An excessive and often insatiable appetite, accompanied by a corresponding great activity of digestion and assimilation, is a fundamental characteristic, and an almost pathognomonic symptom. Organic life appears, in this disease, to stand in an inverse ratio to animal or intellectual life.

Although, according to our view, cerebral congestion is not the immediate, proximate, or initial cause of general paralysis, it nevertheless participates, in a striking manner, in developing a host of consecutive phenomena, which tend singularly to aggravate this disease, and to hasten its fatal termination, inducing chronic or acute inflammation of the membranes, or substance of the brain. The stagnation of the blood in the capillaries opposes the absorption of the serosity, and gives rise to mechanical serous effusions. Thus, whenever, a chemical analysis of the blood of paralytic insane patients manifests a diminution of fibrin, and more especially an augmentation of the globules, we should not hesitate, in the first place, to subject the patients to a very strict vegetable diet; and, in the next, to have recourse to venesection, whatever may be advanced to the contrary by M. Erlenmeyer. Here the question arises, Can hematology explain, more or less perfectly, the mode of formation of the false membranes found in the arachnoid of paralytic insane patients?

Pathological anatomists are at issue regarding the origin of these products. Some maintain that the pseudo-membranes in question are the effect of irritation, determined by the sanguineous effusion in the meninges, or the result of arachnitis, independently of any kind of meningeal hæmorrhage. Others—among whom are MM. Baillarger and Aubanel—refer them to the pre-existence of a meningeal apoplexy, considering them as a pure and simple transformation of the coagulable portion of the effused blood, and not as the solidification of a plastic lymph, effused by the inflamed serous membrane. "It follows," says M. Aubanel, "that as fibrin alone can be organized into false membranes, all the parts of the blood which are capable of being carried away in sanguineous effusions of the arachnoid might be wholly absorbed, and that there might be nothing remaining but the fibrinous portion, which will be transformed into a product perfectly similar to that originating from a primitive fibrinous exhalation. This is actually what happens at a more advanced stage of the development of the false membranes of which we are speaking; for when all traces of blood have disappeared, there remains no differential character in their appearance, or even in their structure, by which they may be distinguished from false membranes having a different origin."*

Before we can admit, with M. Aubanel, that the false membranes of the arachnoid are the simple transformation of the fibrin of the blood effused in the serous membrane, we must first ascertain the condition in which this fibrin exists in the blood, not by means of the scalpel, but by submitting it to a physical inspection, and a chemical analysis. The fact that the blood of persons subject to congestions and hæmorrhages does not easily coagulate, is one that scarcely ever has been called in question; and this fact admits of an easy explanation, since we know, from the researches of MM. Andral and Gavarret, that in persons having this tendency, the fibrin usually undergoes an absolute or a relative diminution. Our own chemical analyses of the blood of paralytic insane patients have yielded perfectly identical results. On the other hand, the blood effused in these cases into the arachnoid varies from a quarter of an ounce to an ounce and a half, its ordinary maximum. According to these data, therefore, if in insanity, accompanied by general paralysis, 1000 grammes of blood yield 2·7 as the mean number for fibrin, as established by our chemical analyses, 45 grammes of blood effused in the arachnoid must

* Des Fausses Membranes de l'Arachniöde chez les Aliénés. (Ann. Médico-Psychol., Sept. 1843, p. 210.)

necessarily yield only 0·03 of fibrin, and here it must be remarked that I do not take the minimum, or even the mean, but the ordinary maximum given by M. Bayle. How can we suppose that so minute a quantity of this principle should have the power of being transformed and organized in such a manner as to produce a false membrane, which is usually equal in thickness to that of the pleuræ or of the dura mater, and which extends over a considerable portion, if not over the whole convexity of one hemisphere?

Chemical analysis is, therefore, unfavourable to the opinion of those authors, who regard sanguineous effusions between the meninges as the origin, and a sufficient cause, of the formation of the false arachnoidal membranes, whilst it is by no means opposed to the views of those pathological anatomists who consider these products as the effect of a secretion, which they attribute to the coagulation of plastic lymph, or, in other words, to the fibrin exhaled from all the membranes, and especially the serous ones, under the influence of an inflammatory condition.

The facts of any acute phlegmasia being always accompanied by an excess of fibrin in the blood; and of a relative or absolute diminution of fibrin being found in the blood of the paralytic insane, are not sufficient evidence to prove the impossibility of an inflammatory condition in the meninges in these cases. The researches of M. Andral have proved that there is no excess of fibrin in the blood prior to the manifestation of acute inflammation, or before the development of any artificial inflammation induced by means of a blister. Inflammation may, therefore, occur in any part, independently of all influences exercised by the previous condition of the blood. If the augmentation of the fibrin be merely a simple phenomenon, progressing simultaneously with the inflammation, and not the cause from whence it arises, there is no reason that the diminution of this principle of the blood should offer any obstacle to the development of meningitis in the paralytic insane, or, consequently, that it should prevent the formation of false membranes, originating in and depending pathogenically exclusively on this inflammation. This meningitis, which has almost always a chronic character, may be primary and give rise to false membranes, which in no way coincide with the existence of meningeal apoplexy; but as pseudo-arachnoid membranes, independently of all sanguineous effusion, are very uncommon, there is reason to believe that the inflammatory condition is most ordinarily consecutive, and is usually determined by the presence of this sanguineous effusion.

Can the spontaneous diminution of the albumen of the blood be one of the causes of cerebral dropsy? Does its influence, combined or not, on the one hand, with that of the chronic inflammation of the meninges, and, on the other, with the influence of the excessive pressure against the walls of the vessels by an embarrassed circulation and by a stagnation of blood in the brain, sufficiently explain this serous effusion under consideration? We know, in physics, that where two fluids of a different nature are separated by a membranous partition, they will both traverse this barrier; and whilst a double current of imbibition will be established, as M. Magendie has shown, the more viscid fluid will attract the other, which, from its lesser viscidity, is better able to permeate through the intervening partition. The celebrated professor of the College of France, who has applied these facts to human pathology, has attempted to make them the basis of a mode of treating encysted dropsy of the ovaries, by modifying these tumours, by the aid of irritating injections, in order that the exhalent surfaces may be brought in contact with a less thick serosity, and one, consequently, which may be more easily absorbed by the vessels of the cyst. The blood which experiences a very considerable diminution in the number of its albumen evidently loses its viscidity. It is less thick when compared to the respective density of the other ambiant fluids, and presents, therefore, all the physical conditions for exosmosis. Nothing would here seem to oppose the filtration of a considerable portion of its serosity through the pores of the vessels, unless we concur in the conjectures of M. Andral, when he asks—"Does the water of the blood flow easily in the capillary vessels, when, being less charged with albumen, it becomes less unctuous, and may therefore, perhaps, flow less easily over the inner surface of the vessels? If such be the case, the diminution of the albumen in the serum of the blood would have the effect of rendering the passage of the fluid through the small vessels more difficult, and, consequently, in relation to its immediate cause, there would not be any such great difference between the dropsy which succeeds an organic disease of the heart or liver, and that which supervenes on the diminution of the number of the albumen of the blood."

I might here employ an argument, based on comparative pathology, in refutation of any objections that may be advanced against these hypotheses, on the ground that, in general paralysis of the insane, we rarely meet with œdema, properly so called, anasarca and ascites, for it may be observed, that in sheep who have flukes in the biliary ducts, and where the dropsy is regarded as the consequence of the diminution, proved by MM. Andral and Gavarret to exist in the albumen of the blood, the infiltration is only manifested in the conjunctiva, and in the soft tissues surrounding the lower jaw, while it is only in cases where the affection is very far advanced that effusions of any extent occur in the serous cavities. I would further advance a very solid argument. Exclusion is as illegitimate in humorism as in solidism. The tenuity of the blood necessarily requires another condition to produce dropsy, consisting, according to the admission of Henle, in a certain relaxation of the pores of the vascular walls. According to this writer, the laxity of tissue is induced by atony, arising probably from a direct paralysis of the nerves—a defect in nervous influence, from stagnation of the blood in the vessels, or from the too great pressure on the vascular walls, by which the fluid is forced through the pores. In general paralysis of the insane, independently of a dynamic primary lesion, there is very frequently a permanent impediment of the circulation within the brain. In adopting the eclectic theory of Henle, we easily understand how it occurs that the serous effusion, instead of taking place in the cellular tissue, in the peritoneum, &c., occurs in the cavity of the arachnoid, in the pia-mater, and the lateral ventricles.

It is, therefore, very probable, from the preceding considerations, that the spontaneous diminution, and the insufficient formation of the albumen of the blood, are the immediate causes of a certain number of the cerebral dropsies which occur in the paralytic insane. However this may be, I think, with M. Bayle, that in these patients the exhalation of the serosity in the cavity of the arachnoid, the serous infiltration of the pia-mater, and, above all, the same kind of effusions in the lateral ventricles, all tend, by the pressure they exercise on the brain, to increase the paralysis, and the enfeeblement of the intellectual faculties. The following fact may be advanced in support of this opinion. I recently bled an insane paralytic patient, whose case is not included in the sixteen that I have already given. The disease had made rapid progress. The paralysis was so considerable, that walking and standing were alike impossible; so decided a degree of enfeeblement of the understanding, that there was almost entire absence of attention and memory—in fact, an actual state of stupidity. On analysing the blood, I found the number of the globules below its mean proportion (119), the fibrin in its normal quantity, and a diminution of the organic matters of the serum. After having, for about a month, taken large doses of aloes three times in the week, which were followed on each occasion by an abundant evacuation of liquid stools, the patient experienced a most marked amendment. Instead of his former stupidity, he evinced an aptitude to converse; and instead of remaining entirely motionless in his bed, he at length became able to stand upright, and walk alone. In this case, the compressure of the brain could not have depended on a sanguineous congestion, since the fibrin of the blood was not diminished, and the globules were below their physiological mean. If, however, there was cerebral compressure, it must rather have been occasioned by an accumulation of serosity, whose removal must have been influenced by the alvine secretions occasioned by the aloes. Here a very rational therapeutic principle would seem to emanate from this pathogenic induction, for the employment, in analogous cases, of purgatives instead of venesection; for the former means have not, at any rate, the disadvantage presented by the latter, of depriving the blood of its globules (the number of which is below the mean), and consequently producing anæmia, which is almost as likely as cerebral congestion to aggravate the paralysis.

Far from proving detrimental to pathological anatomy, chemistry may alike aid and control its application. With equal claims to legitimacy, these two means of investigation may be regarded as kindred lights, which serve mutually to illuminate one another.

### *Resumé.*

From this investigation we arrive at three classes of conclusions:—

1. Chemical facts.
2. Pathogenic inductions.
3. Therapeutic inductions.

### *Chemical Facts.*

1. In general paralysis of the insane, the quantitative analysis of the blood presents very variable results.

2. Augmentation of the globules (the venous crasis of the Germans) exists in the majority of cases. This principle of the blood only remains within its normal proportions in a very small number of cases. It falls below these limits in a still smaller number.

3. The fibrin remains within its physiological limits in the greater number of cases. It exhibits an absolute diminution in a certain minority. It rises above those limits (the fibrinous crasis, the hyperinosis of the Germans) in a still smaller minority of cases.

4. The solid matters of the serum, both organic and inorganic, remain within their normal proportions in the majority of cases. It is only in a very small number that they rise perceptibly above their physiological mean.

5. The organic matters of the serum, of which albumen constitutes so large a proportion, diminish appreciably in rather less than one-third of the cases.

6. The water only exceeds its mean proportion in a very small majority. It falls below it in a considerable minority of cases.

*Pathogenic Inductions.*

1. The augmentation of the globules (venous crasis,) and the absolute diminution of the fibrin (hypinosis), are either, independently of one another, or simultaneously and conjointly, the cause of cerebral congestion, which plays so important a part in the etiology of the general paralysis of the insane.

2. Cerebral congestion is a principal condition, but not the controlling cause of the origin of general paralysis. It is, on the contrary, the proximate or direct cause of the secondary phenomena of this disease.

3. The augmentation of the globules, instead of being inherent in the nature of general paralysis, depends on several purely contingent conditions, such as the male sex, a sanguineous temperament, the strength of the constitution, age, mode of life, voracity, and activity of digestion and assimilation.

4. The diminution of the globules occasionally gives rise to convulsive movements and attacks of catalepsy.

5. The augmentation of the fibrin is frequently associated with epileptic attacks, and several other symptoms of acute inflammation of the brain or its membranes.

6. The false arachnoidal membranes are the result of a coagulation of plastic lymph, secreted by an inflamed surface, and not of the transformation, or the pure and simple organization, of fibrin contained in the blood effused amongst the meninges.

7. The spontaneous diminution of albumen has probably some share in the formation of the more or less considerable serous effusions, which so frequently compress the brain in the last periods of general paralysis.

*Therapeutic Inductions.*

1. Venesection and a moderate vegetable diet are the most rational and efficacious means of preventing the development of cerebral congestion in the paralytic insane, and of combating it when once established.

2. In those cases in which it is supposed that pressure on the brain has been occasioned by an accumulation of serosity, and where an analysis of the blood manifests a tendency towards the diminution of the globules, purgatives and not venesection must be employed.

